# Supplementary figures and images for: Metagenomic analyses of 7000 to 5500 years old coprolites excavated from the Torihama shell-mound site in the Japanese archipelago
Source: PLoS One. 2024 Jan 24;19(1):e0295924. doi: 10.1371/journal.pone.0295924 (PMC10807776; doi:10.1371/journal.pone.0295924)

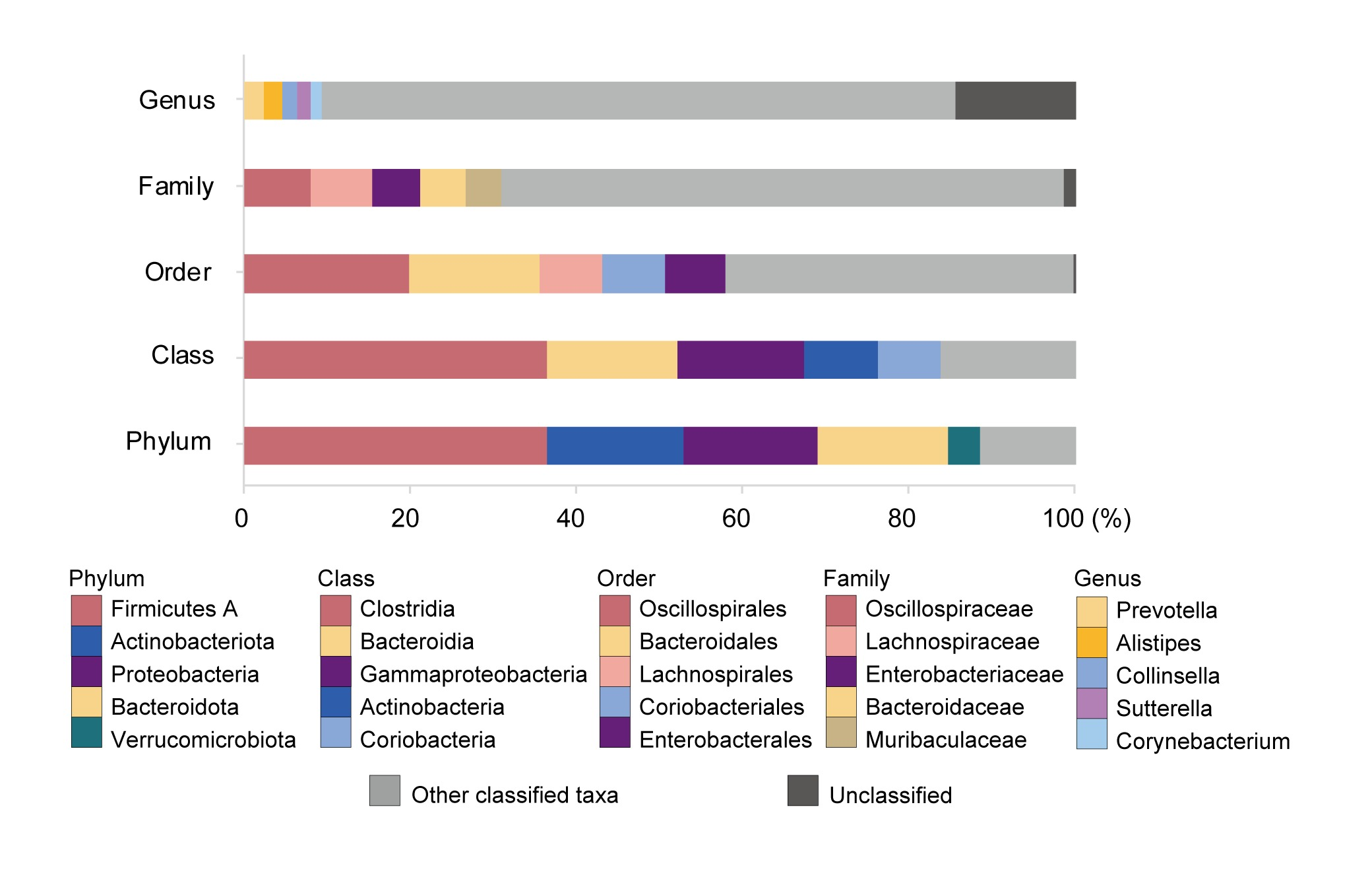

Supplement: S1 Fig — Bar charts show the taxonomic classification of detected gut bacteria and proportion of each bacterial groups. The ratio was calculated based on the number of aligned reads. The colored bars indicate the top five abundant classifications. The light gray bar contains reads of all other known bacterial families, and the dark gray bar contains reads of unclassified bacteria. (TIF) [file pone.0295924.s001.tif]
