# Supplementary material for: Metagenomic analyses of 7000 to 5500 years old coprolites excavated from the Torihama shell-mound site in the Japanese archipelago
Source: PLoS One. 2024 Jan 24;19(1):e0295924. doi: 10.1371/journal.pone.0295924 (PMC10807776; doi:10.1371/journal.pone.0295924)
Supplement: Attachment — Submitted filename: Respponse to Reviewers.docx [file pone.0295924.s012.docx]

December 1, 2023

Yanpeng Li, Ph.D.

Academic Editor, PLOS ONE

Dear Dr. Li;

Thank you for your letter providing positive feedback on our paper “Metagenomic analyses of 7000 to 5500 years old coprolites excavated from the Torihama shell-mound site in the Japanese archipelago” by Nishimura *et al*. We have diligently addressed reviewer’s comments point by point, as outlined below. We trust that the revised version now meets the standards of PLOS ONE.

We are grateful for your insightful and constructive comments, and we have dedicated considerable efforts to incorporating them into our work. With these revisions applied to our final manuscript, we are resubmitting it. We sincerely appreciate your continued consideration of our paper and eagerly await a final decision. Thank you once again for your time and expertise in reviewing our work.

Best wishes,

Ituro INOUE M.D.

Human Genetics Laboratory, National Institute of Genetics, Mishima, Shizuoka, Japan

E-mail address: itinoue@nig.ac.jp

**Response to Reviewers**

**To Reviewer #2**

*This is my 2nd time to review this paper. Again, this is an interesting work, but major concern remains. No human genome construction cannot convince the difficulty for recovering viral genomes that are much shorter – e.g., the work cited in this study (ref 13): https://doi.org/10.1038/s10038-020-00841-6.*

**Reply:**

We express our gratitude to the reviewer for providing numerous critical and valuable comments that significantly improve the manuscript. The manuscript has been thoroughly revised in accordance with the insightful suggestions.

We acknowledge the challenges associated with conducting viral discoveries using our highly fragmented DNA data. In this instance, we have presented the results of aligning these ultra-short reads to reference viral genomes, suggesting the potential existence of these viruses. It is crucial to emphasize that, as of now, we have not definitively identified specific viruses in our coprolite samples.

***Some specific comments:***

*1. Line 100: 2-5 mg or 2-5 g? It’s confusing later “0.08-0.1g” was described at Line 106.*

**Reply:**

2-5 g was accurate, and we have rectified the manuscript. We apologize for the error in writing.

*2. Line 102: Will UV light damage DNA, thus impact mapping?*

**Reply:**

The methodology is a standard for extracting ancient DNA. UV light has the potential to degrade and deactivate DNA, posing a risk of contamination on the sample surface. It is considered that the impact of UV light may not extend to ancient DNA located in the inner part of the samples. For example, Farrer et al. (2021) utilized dental calculus samples and evaluated various ancient DNA extraction protocols, including UV irradiation. Their findings revealed that both EDTA pre-digestion and a combined treatment involving UV irradiation and 5% sodium hypochlorite immersion were effective in reducing the proportion of environmental taxa and increased the abundance of oral taxa compared to untreated samples.

*3.Line 107: Can you provide the numbers for DNA amounts?*

**Reply:**

We revised the sentence and included the DNA amounts as follows: Four DNA extracts from TH55, TH58, TH62, and TH74 containing 5.80 ng, 4.46 ng, 15.54 ng, and 5.36 ng of DNA, respectively, were proceeded to library preparation for metagenomic sequencing.

*4.Line 111: Excluding what size of fractions?*

**Reply:**

Since we conducted size selection with x0.9 volume of AMPure XP Beads, libraries exceeding 250 bp (with an insert size of 130 bp) in total length were eliminated.

*5. Line 139: Why not using the new ICTV taxonomy?*

**Reply:**

Despite the recent abolition of the major families of Caudovirales last year, numerous virome studies continue to rely on the previous viral taxonomic classifications (e.g., Gregory et al. 2020). In order to maintain consistency with earlier reports, we have chosen to adhere to the old taxonomic classifications.

*6. Line 190-180: Is this expected or surprising?*

**Reply:**

While we are not entirely sure which specific sentence you are referring to, we believe you might be alluding to the sentence containing ‘Although sequence variations were not detectable, due to the low depth of alignments, there might be similar or closely related viral species with the present-day gut viruses as those in the gut of Jomon people.’ If so, it was not so surprised by the fact the presence of modern gut bacteria in the gut environment of ancient individuals (e.g., Wibowo et al. 2021, Rampelli et al. 2021.) These gut bacteria could potentially serve as hosts for phages, therefore similar modern gut viruses might exist in the guts of ancient people. Evidently, there is a possibility of distinct viruses existing in the gut of ancient individuals. Further studies will be helpful in uncovering and understanding these potential differences.

*7. Line 193: highly, do you mean well preserved? Then how do you know they are preserved well or not?*

**Reply:**

That is right. We changed the word from ‘highly’ to ‘well.’

To further examine and analyze the new coprolite samples, several methods can be employed for checking preservation conditions, including macroscopic identification of food remains, confirming the extracted DNA amount, and conducting DNA sequencing using a relatively low-throughput instrument such as the Illumina MiSeq platform. The microscopic identification findings could be combined with archaeological data pertaining to pollen or food items recovered from the excavation sites. In DNA sequencing data, the longer length and ancient DNA damage pattern observed in reads originating from the host human, food remnants, bacteria, and viruses could potentially act as indicators of favorable preservation conditions. Once the preservation condition of the DNA is confirmed through screening with the MiSeq platform, subsequent sequencing can be carried out using a high-throughput instrument like the NovaSeq platform to obtain a substantial volume of reads.

*8. Table 2 and 3: Coverage% - ‘%’ was missing in Table 2. What does ‘*’ mean? How much coverage is enough to support a presence of the tested viruses in your samples?*

**Reply:**

Thank you for bringing this point to our attention. We have updated the column name from 'Coverage (1<x)' to 'Coverage% (1<x)' as suggested. Additionally, we have provided an explanation for '*' in the context of using deduplicated reads. In Table 2, where the units of length were absent, we have now included the unit 'bp' and rectified the typo from 'PMD' to 'PMDS.' We have also added the explanation for '*' in all supplementary tables.

Regarding coverages, we were unable to specify the required coverage percentage, so we incorporated all available data. Given that previous studies have identified microbial sequences with at least a few mapped reads (e.g., Rampelli et al., 2021), we also presented viral species with a minimum of one read for PMDS ≥ 3.

*9. Line 229-230: If you map the modern human metagenomes to the giant viral genomes, won’t you also get the mapping like you showed in the tables?*

**Reply:**

We have acquired mapping results of the giant viral genomes based on modern feces metagenomic data (e.g., PRJEB13870) which the average alignment length was approximately 20 bp. It is noteworthy that modern human metagenomic data differs from ancient coprolites in that the DNA does not undergo postmortem degradation. Consequently, in the case of modern human metagenomic data, ultra-short reads with alignment lengths shorter than 30 bp should be excluded during the filtering process, as they are considerably shorter than human DNA. In contrast, in our coprolite samples, human DNA was highly degraded, and as a result, ultra-short reads were retained.
